# Ecological and evolutionary significance of a lack of capacity for extended developmental arrest in crocodilian eggs

**DOI:** 10.1098/rsos.171439

**Published:** 2017-12-20

**Authors:** Sean A. Williamson, Roger G. Evans, S. Charlie Manolis, Grahame J. Webb, Richard D. Reina

**Affiliations:** 1 School of Biological Sciences, Monash University, Clayton, Victoria 3800, Australia; 2 Cardiovascular Disease Program, Biosciences Discovery Institute and Department of Physiology, Monash University, Clayton, Victoria 3800, Australia; 3 Wildlife Management International Pty Limited, PO Box 530, Karama, Northern Territory 0813, Australia; 4 Research Institute for the Environment and Livelihoods, Charles Darwin University, Darwin, Northern Territory 0909, Australia

**Keywords:** embryonic arrest, hypoxia, estuarine crocodile, nesting

## Abstract

Hypoxia within the oviducts maintains embryonic arrest in turtles at the pre-ovipositional stage, which expands the timeframe over which nesting can occur without compromising embryo survival. The arrest can be extended post-oviposition through incubation of eggs in hypoxia. We determined whether crocodilian embryos have this same capacity. We also tested whether increased oxygen availability during incubation alters hatching success. We incubated freshly laid saltwater crocodile (*Crocodylus porosus*) eggs (*N* = 83) at 32°C in one of five treatments; control (normoxia; 21% O_2_), 3-day and 6-day hypoxia (1% O_2_), or 3-day and 6-day hyperoxia (42% O_2_). Incubation (approx. 82 days) was then completed in normoxia. There was a significant effect of treatment on survival of embryos through to hatching (*p* < 0.001). The hypoxic treatments resulted in almost no hatching (6.7% and 0% survival for the 3- and 6-day treatments, respectively), while the hyperoxic and control treatments resulted in normal to high hatching success (86.6%, 100% and 64.2% for the control, 3- and 6-day hyperoxic treatments, respectively). Unlike turtles, hypoxic incubation of crocodile eggs failed to delay development. Our results provide the first experimental evidence that, unlike turtles, crocodiles do not exhibit embryonic arrest when incubated under hypoxic conditions immediately following oviposition. An absence of embryonic arrest is of ecological and evolutionary significance, as it implies that crocodilians lack an ability to avoid adverse environmental conditions through delayed nesting and that, unlike turtles, embryonic arrest may not be a potential explanation for the lack of viviparity in the order *Crocodylia*.

## 1. Introduction

Turtles and crocodilians exhibit a mixture of K- and r-selected species traits [1,2]. That is, they are all relatively long-lived species, with low adult mortality and take many years to reach maturity (K-selected traits) [3–6]. However, they are all oviparous (egg-laying), produce a large number of small offspring and experience high rates of mortality during the life stages before maturity (r-selected traits) [7–9]. Crocodilians and turtles both lay eggs with relatively undeveloped embryos, having only reached the neurulation or gastrulation stages of development [10–12]. Thus, they must nest on land and lay relatively undeveloped eggs, even though the majority of species are aquatic. However, some extinct archosaurs (the class of reptiles to which crocodilians belong) are thought to have been viviparous and able to sever their connection with the terrestrial environment completely [13]. By contrast, because all crocodilians and turtles must deposit eggs into a nest, the reproductive success of the female and the phenotype of any resulting hatchlings are both heavily influenced by the abiotic and biotic conditions of the nest environment [14–20].

To potentially offset exposure to adverse incubation conditions, turtles are able to improve reproductive success by delaying oviposition until abiotic and biotic conditions are more favourable [21–23]. They do this by arresting embryonic development *in utero* (i.e. pre-oviposition) during the gastrulation stage of development [10,11]. Marine turtles may be able to delay nesting by up to nine weeks [24] and some freshwater species can delay nesting for many months [25]. However, there is evidence that extended periods of embryonic arrest through delayed nesting increases early-stage embryonic mortality in leatherback turtles, which typically have naturally low hatching success [26,27]. Pre-ovipositional embryonic arrest is maintained by the low oxygen availability in turtle oviducts [28] and the arrest is broken by the increase in oxygen availability that occurs when eggs are laid into the nest [28–31]. Around 12 h of normoxia (approx. 21% oxygen) is required to break the arrest in turtles and eggs placed into hypoxia (approx. 1% oxygen) within 12 h of oviposition maintain arrest and are protected from movement-induced mortality [31]. When arrest breaks the embryo transitions from a gastrula into the neurulation stage of development and proceeds to completion [11].

In general, there is limited understanding of the early developmental stages of crocodilians [12], particularly regarding development of eggs while in the oviduct [32], as most developmental studies have been conducted on eggs that were at least a few hours old [12,17,33,34]. It is often difficult to obtain and study freshly laid crocodilian eggs, due to maternal protection of the nesting site. However, we know that unlike eggs of all turtle species investigated to date, crocodilian eggs are laid at the neurulation (i.e. more advanced) stage of development and have already developed 10–20 somites [12], leading to the view that they may not arrest [12,21,35], although there is some disagreement [12,36]. Thus, investigation of whether crocodilians also have the ability to arrest development prior to oviposition is warranted. Extended retention of eggs by crocodilians due to adverse environmental conditions has been reported [12,37,38], supporting the existence of pre-ovipositional arrest. However, there is anecdotal evidence from captive crocodilians that they have limited or no scope for developmental arrest during extended retention of eggs [12]. For example, captive females kept in high densities or without access to appropriate nesting sites will lay eggs into the water. Others have been observed to delay nesting (possibly mediated by elevated corticosteroid levels) but with reduced viability of eggs because the embryos were at a more advanced stage of development and may have already adhered to the shell membrane [12]. Furthermore, in contrast to turtle eggs, chilling (less than 26°C) of crocodilian eggs usually results in embryonic mortality (unpublished data reference in [12]). These apparently contradictory observations have not been investigated experimentally.

Delayed nesting in crocodilians sometimes interferes with the formation of the opaque white spot on the shell, which normally forms on the uppermost part of the egg within 24 h post-oviposition [12,39–41]. The white spot forms as a consequence of the embryo facilitating the movement of water from the albumin into the yolk beneath it, which at the time of laying results in rotation of the yolk bringing the embryo to the top with sub-embryonic fluid beneath it [41]. Continued dehydration of the albumin and the eggshell membrane creates the opaque spot, where the shell and vitelline membranes fuse, with the embryo located under that spot [40,41].

If development continues *in utero* such that vitelline membrane attachment occurs within the oviducts, rather than after oviposition, the yolk cannot rotate and embryos attach at random locations rather than at the top. The sub-embryonic fluid then percolates through the yolk to the top, and in the absence of that bathing fluid, embryos not attached at the top of the egg die within a few days. The presence of sub-embryonic fluid at the time of laying, which can be detected by candling, identifies unfertilized eggs (or eggs with very early embryonic death) [41]. Furthermore, the location of the spot indicates whether the oviposition was delayed to the extent that embryos attached prior to egg laying. Post-oviposition, the rate at which the opaque spot forms and spreads can be altered by changing embryonic developmental rate, for example by altering incubation temperature [17]. Although the formation and spread of the opaque spot appear similar in turtles and crocodilians, we do not know if arrested embryonic development experienced by turtles also occurs in crocodilians or whether they have the same response to hypoxia during development.

An interesting role has been suggested for hyperoxia during development, and some crocodilian farmers incubate crocodilian eggs under hyperoxic conditions (26% O_2_) for reputed benefits to hatchling fitness (N. Stevens 2015, personal communication). Long-term incubation in hyperoxia (30% O_2_) results in faster post-hatching growth of American alligators [42], but there are no reports on the impact of changes in oxygen availability during the early stages of embryonic development in crocodilians when the developmental trajectory may be determined. Whether increased oxygen availability influences the rate at which the opaque white spot forms remains to be determined.

Embryonic arrest has been suggested to constrain the evolution of viviparity in turtles [28,31], which along with crocodilians are among the vertebrate groups to never have evolved live-birth [43]. Given the important ecological and evolutionary implications that embryonic arrest may have, we aimed to determine whether crocodiles might have the ability to arrest development prior to oviposition. To do this, we assessed whether saltwater crocodile embryos are able to survive hypoxia by delaying development, as turtles can. We assumed that if crocodiles do not exhibit embryonic arrest in response to hypoxia immediately after oviposition, they would be unable to delay development during hypoxia, embryos would be adversely affected and survival to hatching would be reduced. We also assessed the impact of hyperoxia on development in order to determine whether it has any impact upon developmental success and hatchling fitness.

## 2. Material and methods

### 2.1. Egg collection

Saltwater crocodile (*Crocodylus porosus*) eggs were collected from three captive females during or within 10 min of completion of oviposition in pens at Crocodylus Park (Berrimah, Northern Territory, Australia). Eggs were quickly candled (i.e. egg contents were illuminated by a torch placed on the side of the egg) to detect sub-embryonic fluid, with any unfertilized eggs discarded.

### 2.2. Oxygen treatments

A set of treatments was used to test how changes in the partial pressure of oxygen (PO_2_) affect embryonic development in saltwater crocodiles. Eggs (*N *= 83; total from the three clutches) were evenly distributed between five oxygen treatments (table 1) after one egg from each clutch was opened at the time of egg collection to determine the approximate embryonic stage according to Ferguson's (1985) 28-stage developmental chronology [12,17]. The remainder of the eggs (*N *= 80) were immediately transferred into airtight Perspex containers (Resi-Plex Plastics, North Geelong, Australia). The eggs were placed on a wire mesh allowing them to sit above approximately 10 ml of water at the base of each box.

**Table 1.** Allocation of saltwater crocodile eggs among experimental treatments (*N* = 83).

treatment	eggs	eggs opened (day post-oviposition)	oxygen concentration and duration of treatment
control	21^a^	5 (3 at oviposition, 1 on each of days 3 and 6)	normal atmospheric oxygen (20.9%), 6 days
hyperoxia-3	16	1 (day 3)	approximately 42% O_2_, 3 days
hyperoxia-6	15	1 (day 6)	approximately 42% O_2_, 6 days
hypoxia-3	16	1 (day 3)	approximately 1% O_2_, 3 days
hypoxia-6	15	1 (day 6)	approximately 1% O_2_, 6 days

^a^One additional egg was removed from the control treatment for analyses as it contained two embryos.

The experimental gases were created using 100% nitrogen for the hypoxia treatment and 42% O_2_ in nitrogen for the hyperoxia treatment (Air Liquide, Australia). Each gas was humidified by bubbling it through a chamber filled with water prior to flowing the gas through each container using the inflow and outflow valves. Gas was administered for 3 min at a flow rate of 8 l min^−1^ for each container. Ambient air was circulated through each container in the control treatment for 3 min. The PO_2_ of gas leaving the outflow valve of each box was monitored using an oxygen sensor (Analytical Industries, Pomona, CA) and a data collection device (Pasco, Roseville, CA). The containers were then sealed and placed in an incubator at 32.0 ± 0.2°C and 100% humidity. The maximum time between oviposition of the first egg in each clutch and the placement of all eggs in their respective treatments was approximately 1 h. The Perspex boxes were re-gassed approximately every 24 h over the treatment period (3 or 6 days).

### 2.3. Egg development and hatching success

Eggs were checked twice daily (morning and afternoon) for the presence and position of the opaque white spot that forms on the shell. At the completion of the 3- and 6-day experimental treatments one egg from each treatment was opened to determine embryonic stage. An extra egg was also opened from the control treatment at 3 days post-oviposition, so that embryonic stage at 3 and 6 days post-oviposition could be determined for all treatments. All remaining eggs (*N *= 74) were then placed into plastic trays and returned to the incubator for completion of development in normoxic conditions (21% O_2_). Any eggs that were determined to have died during incubation (identified by the appearance of fungus or discoloration) were removed and opened to determine embryonic stage at death. Towards the end of the 80-day incubation period, eggs were separated by a tray divider, and checked twice daily (morning and afternoon) for any newly emerged hatchlings. Hatching success was calculated for each treatment as the proportion of hatched eggs of the total number of eggs (excluding eggs that were used for embryo staging).

### 2.4. Hatchling morphology and fitness

Hatchlings were housed individually in plastic trays in the incubator until 2 days post-hatching, allowing absorption of excess yolk, after which the morphology and an index of fitness for each hatchling was derived. Total length (±1 mm), snout vent length (SVL; to the front of the cloaca), head width, limb lengths, maximum belly width and maximum yolk scar width (all ± 0.01 mm) and mass (± 0.1 g) were recorded for each hatchling. Hatchlings were allowed to warm to 32.0 ± 0.2°C for at least 5 min before being subjected to running and swimming tests. The running ability of each hatchling was assessed using a 3 m PVC guttering pipe lined with moist sand (15 cm wide). The swimming ability of each hatchling was assessed using a 5.1 m PVC guttering pipe (15 cm wide) filled with water (10 cm deep). Both PVC guttering pipes were kept level. Timing (to nearest second) of a hatchling commenced as soon as they began moving at one end of the pipe and concluded once it reached the other end. The presence of the researcher at the starting point of the track, gently tapping the side of the gutter at the starting end with a plastic pipe, encouraged a unidirectional response by the hatchling towards the opposite end. Each test was repeated three times, with an interval of at least 10 min between each test. All tests were conducted at an air and water temperature of 32 ± 0.2°C. At the completion of testing, hatchings were placed into raising pens at the farm.

### 2.5. Staging dead embryos

All dead and opened eggs were preserved for staging by injecting approximately 4 ml of 10% neutral buffered formalin into the centre of the egg and then placing the whole egg in a specimen jar filled with 10% neutral buffered formalin. Preserved eggs were later carefully dissected following the methodology described by Webb *et al*. [34] using a compound microscope mounted with a camera (Leica Microsystems Pty Ltd, North Ryde, Australia).

### 2.6. Data analysis

One egg from the control treatment did not hatch and was found to contain twin embryos, so it was excluded from all calculations and analyses. One hatchling failed to internalize its yolk and died shortly after hatching, and was excluded from all analyses of morphometry and fitness. All healthy eggs that were opened and staged (*N *= 9) were also excluded from all calculations and analyses (table 1). The time each egg spent in hypoxia was subtracted from total time since oviposition to calculate aerobic latency (time taken) till white spot formation.

Homoscedasticity and normality of continuous dependent variables were assessed using the Fligner–Killeen and Shapiro–Wilks tests. Between-group differences in continuous variables were assessed using analysis of variance (ANOVA) with treatment group as the independent factor and maternal identity as a random blocking factor. *Post hoc* comparisons were made using Tukey's honest significant difference test. Assumptions of normality and homoscedasticity were violated in the cases of total and aerobic latency till white spot, hatching time, hatchling mass, SVL and total length (*p* < 0.05). Consequently, these data were analysed using Kruskal–Wallis and Nemenyi *post hoc* tests. Between-group differences in hatching success were assessed using Cochran–Mantel–Haenszel (CMH) tests (adjusting for maternal identity) with Bonferroni corrections for pair-wise comparisons. *Post hoc* analysis of hatching success was completed using Bonferroni corrected chi-squared tests with treatment group as the independent variable. Fisher's exact test was used to test for between-group differences in the proportion of embryos that died at either early, mid or late stages of development. Embryos that died between Ferguson's (1985) stages 1 and 10 were classified as early, 11 and 23 as mid and 24 and 28 as late. All analyses were conducted using R software [44]. All values are presented as mean ± s.e. Two-tailed *p *≤ 0.05 was considered statistically significant.

## 3. Results

### 3.1. Opaque white spot formation and embryonic development

**Figure 1.**
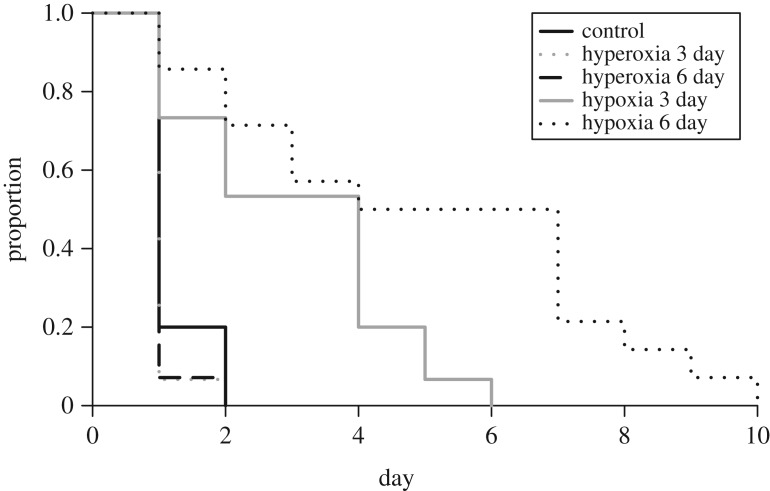
Proportion of saltwater crocodile eggs remaining to form white spots, during the first 10 days after oviposition, among the five treatments. Eggs (*N *= 73) were incubated in either normoxia (control), hyperoxia (42% O_2_) for 3 or 6 days, or hypoxia (1% O_2_) for 3 or 6 days (*n *= 14–15).

**Figure 2.**
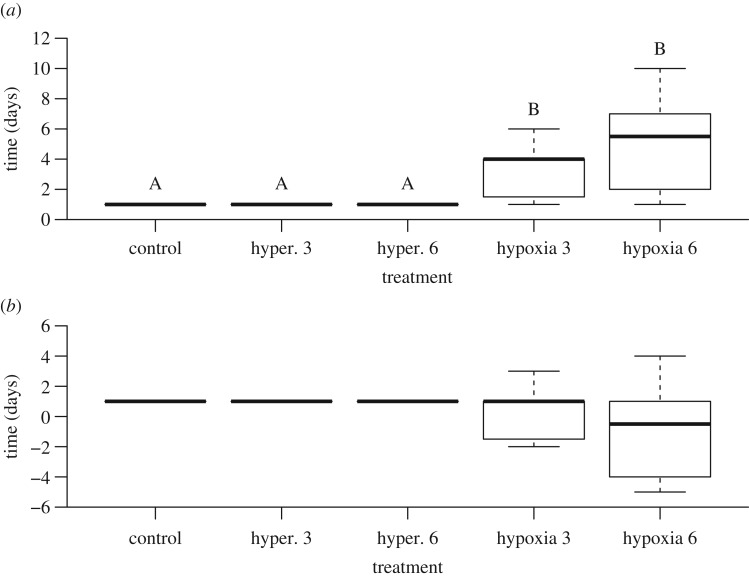
Latency from oviposition to formation of the white spot on saltwater crocodile eggs in (*a*) total time and (*b*) aerobic time. Eggs (*N *= 73) were incubated in either normoxia (control), hyperoxia (42% O_2_) for 3 (hyper. 3) or 6 (hyper. 6) days, or hypoxia (1% O_2_) for 3 (hypoxia 3) or 6 days (hypoxia 6) (*n *= 14–15). Aerobic time is the total time from oviposition excluding time spent in hypoxia. Boxplot centre lines show medians; box limits indicate the 25th and 75th percentiles; whiskers extend 1.5 times the interquartile range from the 25th and 75th percentiles. For (*a*) if the letters above each whisker are the same, latency to white spot formation did not differ significantly between corresponding treatment-groups (Kruskal–Wallis and Nemenyi's *post hoc* test; *p* < 0.0001). For (*b*) there was no significant between-group difference (Kruskal–Wallis and Nemenyi's *post hoc* test; *p* > 0.05).

All eggs formed an opaque white spot on the upper surface of the egg after oviposition. The eggs used were thus all considered to have been fertilized, and there was no indication that any had been subjected to delayed oviposition, because no opaque white spots formed on the sides or bottom of the eggs. However, there was significant between-treatment variation in the latency (time taken) to opaque white spot formation (*H* = 40.05, d.f. = 4, *p* < 0.0001; figures 1 and 2*a*). Eggs from the 3-day hypoxic treatment on average took 45 h longer to form opaque white spots than those from the control treatment (figure 2*a*). Eggs from the 6-day hypoxic treatment on average took 93 h longer than the control (figure 2*a*). Despite these observations being consistent with arrested embryonic development, there was a large variation in latency till opaque white spot formation for both of the hypoxic treatments, with approximately half of the eggs from each treatment forming spots prior to removal from hypoxia (7 of 15 for 3-day hypoxia and 7 of 14 for 6-day hypoxia; figure 1). Once the time spent in hypoxia was accounted for (‘aerobic incubation time’; defined as total time excluding time spent in hypoxia) there was no significant between-group difference in latency to opaque white spot formation (*H* = 8.21, d.f. = 4, *p* = 0.08; figure 2*b*).

The embryonic stage of eggs that were opened differed between the hypoxic and the aerobic (i.e. control and hyperoxic) treatments (table 2). That is, all embryos were at the stage of development expected for their age [12], except the two that were opened from the hypoxic treatments, which were either one stage behind developmental schedule [12] or had not developed at all (table 2).

**Table 2.** Stage of development of saltwater crocodile eggs randomly selected from the various treatments. Embryos (*N* = 9) were staged according to Ferguson's (1985) 28-stage developmental chronology. Eggs were incubated in either normoxia (control), hyperoxia (42% O_2_) for 3 or 6 days or hypoxia (1% O_2_) for 3 or 6 days.

day	control	hyperoxic treatments	hypoxic treatments
0	1, 1, 1	—	—
3	3	3	2
6	6	6	1

In summary, white spot formation was delayed in a non-systematic way by hypoxic incubation, while hyperoxic incubation did not have any detectable impact upon timing of white spot formation. Furthermore, eggs that we opened after removal from hypoxia had embryos that were behind in their predicted developmental schedule.

### 3.2. Hatching, embryonic death and hatchling traits

**Figure 3.**
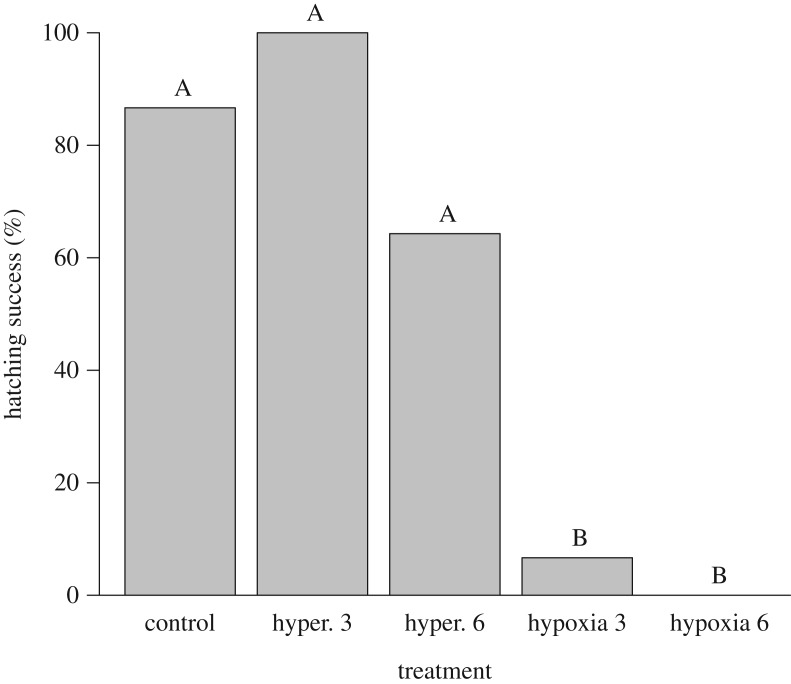
Proportion of saltwater crocodile eggs to hatch (%) after the various treatments. Eggs (*N *= 73) were incubated in either normoxia (control), hyperoxia (42% O_2_) for 3 (hyper. 3) or 6 (hyper. 6) days or hypoxia (1% O_2_) for 3 (hypoxia 3) or 6 days (hypoxia 6) (*n *= 14–15). Following their respective treatments all eggs were incubated in normoxia until hatching. When letters above each bar are the same, there was no significant between-group difference in hatching success (Bonferroni corrected chi-squared test with 10 pair-wise comparisons; *p* ≤ 0.05).

**Figure 4.**
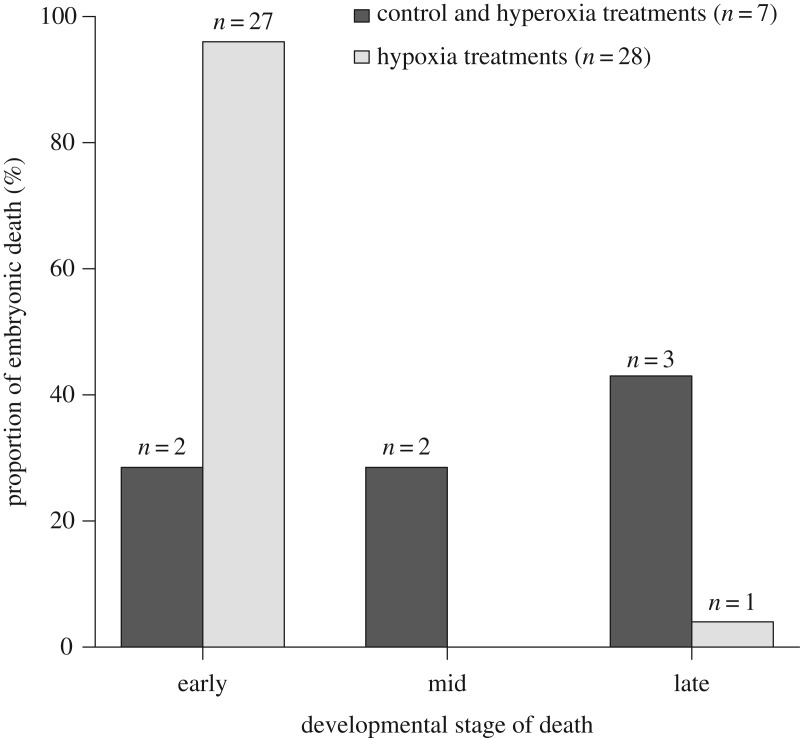
Proportion of embryonic death at three stages of development in saltwater crocodile eggs. Eggs (*N *= 73) were incubated in either normoxia (control), hyperoxia (42% O_2_) for 3 or 6 days, or hypoxia for 3 or 6 days (*n *= 14–15). Following their respective treatments, all eggs were incubated in normoxia until hatching. For this analysis, experimental groups were combined into those in which there was a low incidence of embryonic death (0–35.7% for control and hyperoxia treatments; 7 embryos total) and those in which there was a high incidence of embryonic death (93–100% for both hypoxia treatments; 28 embryos in total). Embryos were staged according to Ferguson's (1985) 28-stage developmental chronology and further classified as either early (stages 1–10), mid (stages 11–23) or late (stages 24–28).

Hatching success varied significantly among the various treatments (*X*^2^_CMH_ = 48.29, d.f. = 4, *p* < 0.0001; figure 3) with no three-way association with female identity (Woolf test *X*^2^ = 0.67, d.f. = 2, *p* = 0.71). The three aerobic treatments (control and two hyperoxic treatments) had greater hatching success (64.3–100%) than the two hypoxic treatments (0–6.7%; figure 3). There was no significant between-group variation in the time taken to hatch (*H* = 1.10, d.f. = 3, *p* = 0.78). All eggs took an average of 81.5 days to hatch (table 3). For eggs that failed to hatch, there was significant between-treatment variation in the proportion of embryos that died at each developmental period (Fisher's exact test; *p* < 0.001). Dead embryos from treatments that had low hatching success (both hypoxia treatments) typically died early during development, whereas those from treatments that had normal hatching success for crocodiles (control and both hyperoxia treatments) died at stages throughout development (figure 4). From the eggs that hatched, there was no significant between-group variation in hatchling morphology or fitness traits (table 3; *p* > 0.05).

**Table 3.** Traits of saltwater crocodile hatchlings from various treatments. Eggs (*N* = 38) were incubated in either normoxia (control), hyperoxia (42% O_2_) for 3 or 6 days, or hypoxia (1% O_2_) for 3 or 6 days. Following their respective treatments all eggs were incubated in normoxia until hatching. Front (F) and back (B), left (L) and right (R) leg lengths (LL) were measured.

	control	hyperoxia (3-days)	hyperoxia (6-days)	hypoxia (3-days)	hypoxia (6-days)	test statistic	*p-*value
no. of hatchlings	13	15^a^	9	1	0	*X*^2^_CMH_ = 42.65	<0.0001
hatching time (d)	81.5 ± 0.2	81.3 ± 0.2	81.8 ± 0.5	82.0	N/A	*H*_(__3,33)_ = 0.78	0.78
mass (g)	77.0 ± 1.1	78.1 ± 0.8	76.4 ± 0.5	80.2	N/A	*H*_(__3,33)_ = 4.40	0.22
SVL (mm)	143.4 ± 1.1	143.9 ± 0.7	140.1 ± 1.6	140.9	N/A	*H*_(__3,33)_ = 4.55	0.21
total length (mm)	306 ± 4	304 ± 1	300 ± 2	299	N/A	*H*_(__3,33)_ = 3.71	0.29
head width (mm)	23.2 ± 0.1	23.4 ± 0.5	23.0 ± 0.1	24.0	N/A	*F*_(__3,27)_ = 1.38	0.37
FRLL (mm)	50.3 ± 0.4	50.2 ± 0.6	50.2 ± 0.4	50.2	N/A	*F*_(__3,27)_ = 0.39	0.77
FLLL (mm)	50.7 ± 0.6	51.3 ± 0.6	50.1 ± 0.5	50.0	N/A	*F*_(__3,27)_ = 2.41	0.21
BRLL (mm)	63.1 ± 0.4	63.8 ± 0.5	63.9 ± 0.6	60.8	N/A	*F*_(__3,27)_ = 0.85	0.54
BLLL (mm)	62.8 ± 0.4	63.2 ± 0.6	63.0 ± 0.5	62.5	N/A	*F*_(__3,27)_ = 0.18	0.90
belly width (mm)	39.0 ± 0.5	39.9 ± 0.4	40.8 ± 0.5	40.4	N/A	*F*_(__3,27)_ = 1.39	0.37
yolk scar width (mm)	4.3 ± 0.3	4.1 ± 0.4	4.9 ± 0.3	4.4	N/A	*F*_(__3,27)_ = 0.97	0.49
swim speed (cm s^−^^1^)	23.0 ± 0.4	22.9 ± 0.3	23.0 ± 0.4	24.9	N/A	*F*_(__3,27)_ = 0.36	0.79
run speed (cm s^−^^1^)	27.8 ± 1.0	28.7 ± 1.0	26.4 ± 1.5	28.4	N/A	*F*_(__3,27)_ = 3.89	0.11

^a^One hatchling failed to internalize its yolk after hatching and died. Subsequently, it was removed from analysis except for hatching time.

In summary, hypoxic incubation resulted in mortality of almost all embryos. Furthermore, the embryos from the hypoxic treatments died at early developmental stages. However, between the hyperoxic and control treatments we found no difference in hatching success or stage of development for dead embryos.

## 4. Discussion

We showed that crocodile eggs cannot survive hypoxia immediately after oviposition in the way that turtle eggs can, suggesting that, unlike turtles, crocodilians may not arrest embryonic development prior to oviposition. We found that hypoxia did not completely delay development, because opaque white spots still formed and some embryos continued to grow while in hypoxia. This result is in contrast to the response shown in turtles, which exhibit pre-ovipositional embryonic arrest [28–31,45–47]. Our results appear to explain why crocodilians that are faced with adverse nesting conditions either oviposit impaired embryos or lay eggs underwater [12]. The ecological implication is that crocodilians have limited capacity to improve reproductive success by delaying nesting during a breeding period of sub-optimal environmental conditions if they have already commenced ovulation. However, there is anecdotal evidence that many females will oviposit their eggs during storm activity on the same day, which suggests that they can hold onto eggs for a period of time, but cannot do so for long periods like turtles can.

Crocodilian eggs require sufficient oxygen immediately after oviposition for development to continue successfully to hatching. Our results show that an extremely hypoxic incubation environment of 1% oxygen (PO_2_ approximately 8 mmHg) for 3–6 days, commencing within 1 h of oviposition, led to embryonic death at early stages of development and so significantly reduced hatching success. This contrasts with what has been found for turtle embryos, which arrest development in hypoxia and usually recommence development once returned to normoxia [29,31,47,48], although some effects on subsequent hatching success have been observed [28–30]. Flooding of nesting habitat inundates crocodilian nests and is a common cause of mortality [49–54]. If this were to occur for 3 days at the start of development of a clutch of eggs, our findings suggest that the hypoxic conditions (less than 1% O_2_ availability in water) created would be lethal for the embryo. Indeed, there was total mortality of alligator eggs that were subjected to experimental flooding for 2 days [55].

We found no detectable impact of hyperoxic incubation on crocodilian development or hatchlings. Hyperoxia for 3 or 6 days after oviposition did not change developmental timing, hatching success or hatchling fitness and morphology. Our findings are consistent with the only other similar experiment reported, in which there was also minimal impact of short-term hyperoxia during early incubation of flatback sea turtle eggs (*Natator depressus*; [30]). However, it has been shown that smaller increases in oxygen availability than we used (30% versus 42% O_2_), but during later stages of development, have positive impacts upon embryonic development and hatchling fitness of American alligators [42]. Atmospheric oxygen availability has fluctuated greatly throughout crocodilian evolutionary history (from about 13% to 31% O_2_) and incubation of alligator eggs in levels of hyperoxia that were experienced have been shown to positively affect development and bone composition, with an optimum at 27% O_2_ [56,57]. No effect of hyperoxic incubation during early development has now been shown (this study, [30]) for two oviparous reptiles (*C. porosus* and *N. depressus*), but early-stage embryonic death is uncommon for both of these species [16,58,59]. However, extended pre-ovipositional embryonic arrest has been implicated in higher proportions of early-stage embryonic death in leatherback turtles [26]. Further, we know that arrest is broken by an increase in oxygen availability [28,29]. In a species with relatively low hatching success, such as the leatherback turtle, it is possible that hyperoxia might stimulate development and reduce the high level of early-stage embryonic death typically found [27]. Therefore, it seems useful to now assess how hyperoxia during early development might affect a species with pre-ovipositional embryonic arrest and high amounts of early-stage embryonic mortality, such as the leatherback turtle [27].

Our findings suggest that a hypoxic environment would not be suitable for safe transportation of crocodilian eggs, because hypoxia did not delay development. Failure of hypoxia to delay development means that embryos would commence development even during transportation and would render eggs susceptible to movement-induced mortality. This contrasts with our results with turtles, where hypoxic incubation delayed development and subsequently protected eggs from movement-induced mortality [31,60]. Sudden jolting of crocodile eggs during transport, between 8 and 12 days post-oviposition, is likely to result in embryonic mortality because the recently formed chorioallantois is fragile. However, data from another study indicate that rotation of a crocodile egg can kill the embryo after it has attached to the shell (approx. 1 day post-oviposition) but prior to adequate development of the respiratory and excretory functions of the allantois [40]. Deeming and Ferguson [61] showed that eggs can withstand 60° rotations, but care should always be taken to prevent unnecessary movement of eggs [12,62]. Therefore, it is prudent that crocodilian researchers and conservationists continue to exercise caution during transport of eggs.

In order to further develop our understanding of the evolution of viviparity, it is useful and interesting to understand why it has not evolved in some particular taxa [43]. We showed that crocodile embryos are not arrested by hypoxia at oviposition. This lack of hypoxia-mediated embryonic arrest would be one fewer physiological constraint on the evolution of viviparity or even facultative oviparity [16,31]. Our finding that eggs fail to develop while incubated in hypoxia, for even as short as 3 days after oviposition, suggests that increased embryonic development *in utero* would require sufficient oxygen availability within the oviducts of crocodilians [63].

Crocodilian embryos could still be susceptible to movement-induced mortality if eggs are retained in the oviduct beyond the normal time for oviposition. Therefore, obligate oviparity (eggs oviposited with an early-stage embryo) may be an evolutionary one-way path that precludes subsequent evolution of advanced development *in utero* and ultimately of viviparity in this taxon [16]. The evolution of some parental care, in the form of nest- and crèche-guarding, may have been important in the evolution of crocodilian embryonic developmental patterns [12,16,64]. Crocodilians may experience less selection pressure to delay nesting because, unlike turtles, they are well-equipped to protect themselves and their nests from most predation.

It has also been suggested that a morphological difference in oviducts between crocodilians and other vertebrates is important for the evolution of their respective developmental patterns [65]. Crocodilians have an ‘assembly-line’ oviducal morphology where each region performs one task such as calcium secretion or eggshell membrane formation while other taxon, such as turtles, are able to perform both functions in the same region [65]. Perhaps this difference may explain why crocodilians do not arrest development, but further investigation of the evolution of reproduction in crocodilians is needed to understand why this almost entirely aquatic taxon has remained oviparous. It is possible that crocodilian oviducal oxygen availability is a constraint on further *in utero* development, as it may be for turtles, and warrants investigation [28].

A possible limitation in our current study results from the maximum of 1 h delay between oviposition and placement of eggs into their respective treatments. Our conclusion that pre-ovipositional developmental arrest may not occur in saltwater crocodiles is based on the assumption that this very brief period of normoxia did not cause the embryos to recommence development if they were in a state of arrest when eggs were laid. However, our assumption is supported by data from green turtles showing that the eggs require at least 12 h of normoxia after oviposition in order for pre-ovipositional embryonic arrest to be broken [31]. The short delay between oviposition and placement of eggs into their respective experimental treatments is difficult to reduce or remove with crocodilians, because of safety concerns for researchers while trying to collect freshly laid eggs from such an aggressive and dangerous animal. Avoiding the elapsed time between laying of the first and last eggs (10–45 min typically), by collecting each egg as it is oviposited, cannot be achieved without considerable risk. Inducing females to lay eggs while restrained is not advisable because it is impossible to determine exactly when the eggs would have been laid if the female was left to nest naturally. Thus, while we think that the brief period of normoxia is unlikely to have influenced the developmental progression of the embryos, we are unable to discount this possibility.

In conclusion, we have provided experimental evidence leading us to conclude that crocodilian embryos probably do not undergo pre-ovipositional arrest. Our observations and conclusion are consistent with the limited anecdotal evidence from farmers and previous researchers [12]. Importantly, it means that unlike turtles, crocodilians have limited capacity to avoid adverse nesting conditions by delaying nesting. The discovery of a presumptive lack of pre-ovipostional embryonic arrest in crocodilians prompts further investigation as to why this predominately aquatic taxon is dependent on obligate oviparity, especially when other aquatic archosaurs were probably viviparous. We found no detectable impact of hyperoxic incubation on crocodilian development and hatchling fitness. We suggest that hyperoxic incubation could be used to improve hatching success in species that arrest development prior to oviposition and experience high levels of early embryonic mortality. The evolutionary implications of the reproductive strategy of crocodilians suggested by our results may be an interesting avenue for investigation in understanding the prerequisites for viviparity.

## Data accessibility

Our data are deposited at Dryad: http://dx.doi.org/10.5061/dryad.nq41m [66].
